# Assessing fidelity to evidence-based quality improvement as an implementation strategy for patient-centered medical home transformation in the Veterans Health Administration

**DOI:** 10.1186/s13012-020-0979-y

**Published:** 2020-03-18

**Authors:** Susan E. Stockdale, Alison B. Hamilton, Alicia A. Bergman, Danielle E. Rose, Karleen F. Giannitrapani, Timothy R. Dresselhaus, Elizabeth M. Yano, Lisa V. Rubenstein

**Affiliations:** 1 HSR&D Center for the Study of Healthcare Innovation, Implementation, and Policy, VA Greater Los Angeles Healthcare System, 16111 Plummer Street (152), Sepulveda, CA 91343-2039 USA; 2 Department of Psychiatry and Biobehavioral Sciences, University of California, Los Angeles, CA USA; 3 HSR&D Center for Innovation to Implementation, VA Palo Alto Healthcare System, Palo Alto, CA USA; 4 Department of Primary Care and Population Health, Stanford University, Palo Alto, CA USA; 5 Primary Care Service, San Diego VA Healthcare System, San Diego, CA USA; 6 Department of Health Policy & Management Fielding School of Public Health, University of California, Los Angeles, USA; 7 Department of Medicine David Geffen School of Medicine, University of California, Los Angeles, USA; 8 RAND Corporation, Santa Monica, CA USA

**Keywords:** Implementation strategy, Fidelity, Patient-centered medical home, Evidence-based quality improvement

## Abstract

**Background:**

Effective implementation strategies might facilitate patient-centered medical home (PCMH) uptake and spread by targeting barriers to change. Evidence-based quality improvement (EBQI) is a multi-faceted implementation strategy that is based on a clinical-researcher partnership. It promotes organizational change by fostering innovation and the spread of those innovations that are successful. Previous studies demonstrated that EBQI accelerated PCMH adoption within Veterans Health Administration primary care practices, compared with standard PCMH implementation. Research to date has not documented fidelity to the EBQI implementation strategy, limiting usefulness of prior research findings. This paper develops and assesses clinical participants’ fidelity to three core EBQI elements for PCMH (EBQI-PCMH), explores the relationship between fidelity and successful QI project completion and spread (the outcome of EBQI-PCMH), and assesses the role of the clinical-researcher partnership in achieving EBQI-PCMH fidelity.

**Methods:**

Nine primary care practice sites and seven across-sites, topic-focused workgroups participated (2010–2014). Core EBQI elements included leadership-frontlines priority-setting for QI, ongoing access to technical expertise, coaching, and mentoring in QI methods (through a QI collaborative), and data/evidence use to inform QI. We used explicit criteria to measure and assess EBQI-PCMH fidelity across clinical participants. We mapped fidelity to evaluation data on implementation and spread of successful QI projects/products. To assess the clinical-researcher partnership role in EBQI-PCMH, we analyzed 73 key stakeholder interviews using thematic analysis.

**Results:**

Seven of 9 sites and 3 of 7 workgroups achieved high or medium fidelity to leadership-frontlines priority-setting. Fidelity was mixed for ongoing technical expertise and data/evidence use. Longer duration in EBQI-PCMH and higher fidelity to priority-setting and ongoing technical expertise appear correlated with successful QI project completion and spread. According to key stakeholders, partnership with researchers, as well as bi-directional communication between leaders and QI teams and project management/data support were critical to achieving EBQI-PCMH fidelity.

**Conclusions:**

This study advances implementation theory and research by developing measures for and assessing fidelity to core EBQI elements in relationship to completion and spread of QI innovation projects or tools for addressing PCMH challenges. These results help close the gap between EBQI elements, their intended outcome, and the finding that EBQI-PCMH resulted in accelerated adoption of PCMH.

Contributions to the literature

- Improving understanding of effective implementation strategies for patient-centered medical homes (PCMH) could facilitate uptake, but existing studies provide insufficient detail about implementation strategies and fidelity to these strategies for PCMH.
- In previous studies, primary care practices using an evidence-based quality improvement (EBQI) implementation strategy demonstrated better achievement of PMCH goals compared with control sites. We identified three core EBQI elements and found that most EBQI sites had high or medium fidelity on at least 2 core elements.
- These findings contribute to gaps in the literature, including measuring/assessing implementation strategy fidelity and identifying effective PCMH implementation strategies.

## Background

The patient-centered medical home (PCMH) model is widely endorsed by professional societies and has been shown to improve quality of care and patient, provider, and staff satisfaction, while reducing costs [1–4]. Transforming primary care toward the PCMH model, however, requires cultural, technical, and clinical innovation [5]. Implementation strategies are methods to promote adoption, implementation, and sustainability of a new practice, and might facilitate PCMH adoption and spread by targeting barriers to change at multiple levels (e.g., external context, within the organization, among professionals, and in the intervention) [6].

Implementation researchers are developing a knowledge base about which implementation strategies are effective for promoting uptake of evidence-based interventions within different contexts, and have argued that assessing fidelity to implementation strategies (e.g., the extent to which intended implementation strategies are used) is critical [7, 8]. Some studies describe or evaluate PCMH implementation [9–12], but few document or assess fidelity to PCMH implementation strategies, and fewer still assess the relationship between the strategies and their intended outcomes [13–15]. Most do not include sufficient detail to support replication of the strategy, and few address use of implementation strategies to promote PCMH in large, integrated healthcare systems like the Veterans Health Administration (VHA) [16].

In 2010, VHA embarked on nationwide PCMH transformation called Patient Aligned Care Teams (PACT) [17, 18]. In parallel with VHA’s implementation effort, we introduced evidence-based quality improvement (EBQI) as a multi-faceted implementation strategy to improve PCMH implementation in one VHA region [19, 20]. Our ongoing impact evaluation has shown that EBQI-PCMH sites, in comparison to control sites, experienced accelerated achievement of PCMH goals, including decreases in ambulatory care visits, increases in non-face-to-face visits, lower primary care provider burnout, and larger improvements in patient-provider communication [21–23].

**Fig. 1:**
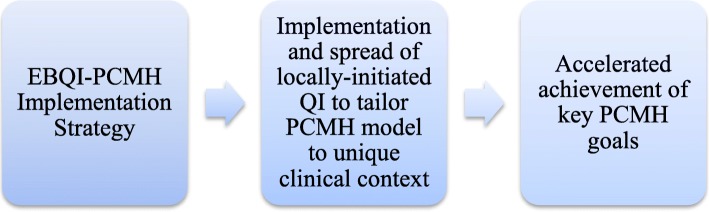
EBQI-PCMH promotes organizational change through implementation and spread of practice-level systematic quality improvement.

The primary outcome of EBQI-PCMH is development and spread of locally initiated primary care QI innovation projects directed at achieving adherence to the PCMH model (Fig. 1). These projects reflect three main inputs. First, they are proposed by frontline clinicians and staff and selected by regional and executive leaders through a priority setting process [19]. Second, QI project development and completion are supported by an ongoing collaborative that features technical support by health services researchers. Third, development of the QI projects is informed not only by PCMH literature, but by targeted evidence specific to each QI project from a responsive evidence review [24] and current local data [20]. The QI innovation projects are the short-term outcomes of EBQI-PCMH, and are the hypothesized link between EBQI-PCMH and longer-term PCMH outcomes. The investigation reported here tests the link between fidelity to 3 core EBQI elements, or inputs (leadership-frontlines priority-setting; ongoing access to technical expertise, coaching, and mentoring in QI methods through a QI collaborative; and data/evidence use to inform QI), and the EBQI outcome of completed, spreadable QI innovation projects for supporting PCMH in VHA.

In addition to assessing the EBQI outcome of successful QI innovation project completion and spread, this paper addresses the critical role of partnership between healthcare clinical leaders and health services researchers [25, 26], which provided the substrate for EBQI-PCMH. Our fidelity measures focus on the extent to which clinical participants engaged in EBQI activities, but do not describe or assess the role of health services researchers. Understanding how health services researchers supported and engaged clinical participants, what clinical participants found valuable, and what the health services researchers could have done better is critical to understanding variations in EBQI-PCMH fidelity. For understanding the role of health services researchers in the clinical-researcher partnership and achievement of EBQI-PCMH fidelity, we analyze key stakeholder qualitative interview data.

Using data abstracted from project records and key stakeholder interviews, the objectives of this study are the following: (1) to develop measures and assess variations in fidelity for three core EBQI-PCMH implementation strategy elements, (2) to explore the relationship between EBQI-PCMH fidelity and its primary outcome (implementation and spread of locally developed and initiated QI innovation projects), and (3) to assess the role of health services researchers to understand variations in EBQI-PCMH fidelity.

### VHA PCMH implementation and the EBQI-PCMH implementation strategy

VHA’s standard national PCMH implementation strategies included a national mandate describing the PCMH elements to be implemented (e.g., PCMH core principles, clinic restructuring, new staffing model, and guidance on new roles and responsibilities), increased funding for primary care staffing, regional learning collaboratives and training centers, new performance measures, and an online toolkit [18, 27–29]. A few studies during early implementation described local efforts to implement PCMH and implementation barriers and facilitators [17, 30–32], and a national evaluation developed a measure to rank clinics on PCMH model fidelity [33]. These studies show considerable variation in VHA’s PCMH implementation, but only one assessed fidelity to an implementation strategy (e.g., a learning collaborative) and association with performance [16].

EBQI-PCMH was developed by the Veterans Assessment and Improvement Laboratory (VAIL) and funded by the VHA Office of Primary Care. Based on EBQI interventions developed by Rubenstein and colleagues [34–36], EBQI was effective for improving uptake of evidence-based clinical practices such as collaborative care for depression [34, 37], supported employment [38], cultural competency for healthcare staff gender sensitivity [39], and VHA’s PCMH implementation [21–23]. Rubenstein et al. have previously described the theoretical basis underlying EBQI-PCMH [19]. The intervention key features were derived from literature on PCMH implementation challenges and theories of organizational change [40–43], clinical quality improvement [44–46], complex adaptive systems [5], and diffusion of innovation [47, 48]. Rubenstein et al.’s logic model illustrated hypothesized relationships between EBQI-PCMH intervention features (e.g., organizational structures and the clinical-researcher partnership activities) and short, intermediate, and long-term outcomes. Local implementation and spread of successful QI innovation projects is a short-term outcome of EBQI-PCMH, hypothesized to result in intermediate and long-term outcomes such as improved achievement of PCMH goals and metrics, workforce job satisfaction, and improved patient experiences [19].

In previously published work, we evaluated EBQI-PCMH organizational structures [20]. Here we derive the three core elements of the EBQI-PCMH implementation strategy from the clinical-researcher partnership activities conceptualized by Rubenstein et al., which include the following: (1) regional consensus-based priority setting that includes leaders and frontlines; (2) communication, collaboration, and coaching; and (3) use of evidence and data (including formative feedback) to inform locally initiated innovation [19]. To arrive at the three core elements, we mapped these activities to VAIL project administrative records for empirical substantiation. Table 1 provides a more detailed description of the core elements, which include the following: (1) a leadership and frontline (i.e., top-down, bottom-up) priority-setting process for focusing QI efforts; (2) ongoing technical expertise and coaching/mentoring in QI methods by health services researchers, delivered through a QI collaborative; and (3) the use of data and evidence to inform QI efforts with project management support provided by internal coordinators.

**Table 1:** Description of EBQI-PCMH core elements and criteria for assessing EBQI-PCMH fidelity

EBQI-PCMH core elements	Fidelity assessment criteria
**Leadership and frontlines** (**e.g.**, **top-down**, **bottom-up**) **priority-setting process for focusing QI efforts** ● Four rounds of priority-setting (1/year, 2011–2014), engaging interdisciplinary regional and healthcare system leaders, frontline providers, staff ● Quality councils (including Veteran patients) and across-site workgroups submitted 2-page project proposals for QI projects for review/approval ● Regional Steering Committee (multidisciplinary regional and local healthcare system executive leaders) reviewed/rated, discussed at in-person meeting, and re-rated ● Seven to 8 highest rated projects approved per round received seed funding, support from VAIL HSRs, completed progress and final reports on their QI projects	Sum of number of proposals submitted and number approved: high = 8 or more; medium = 4–7; low = 1–3
**Ongoing technical expertise and coaching/mentoring in QI methods by HSRs**, **delivered through a QI collaborative** ● Quality council leaders participated in bi-weekly support calls with two or three HSRs (87 calls, 2010–14) ● HSRs provided one-on-one mentoring/support for workgroup projects by joining the individual workgroup project meetings ● Semi-annual in-person conferences (7 total, 2010–14), attended by the QI teams, HSRs, regional and healthcare system leaders, patient representatives, frontline providers and staff at EBQI-PACT sites, and subject matter experts. ○ Plenary sessions providing training in QI and the PCMH model, workshops on PCMH topics, and presentations by QI teams on QI projects ○ Formative feedback presentations of PC practice-level data from the PCMH evaluation, including provider and staff burnout and patient satisfaction ○ “Round table” sessions for QI teams to plan and strategize PCMH improvement	Bi-weekly phone calls: high = participated in 75% or more with an average representation of 2 or more people per call; medium = participated in 75% or more with an average participation of 1–2 representatives per call; low or none = participated in less than 75% with an average participation of less than 1 representative per call Bi-annual in-person learning sessions: high = 10 or more participants per learning session; medium = 5–9 participants; low/none = less than 5 participants
**Use of data and evidence to inform QI efforts with project management support provided by internal coordinators** ● 5 HSRs (LR, SS, SV, JD, BS) supported by 2 statistical analysts (AL, MW) and 5 program support staff (NS, AS, ALH, NS, DE) ● 1 FTE internal coordinator for each of 3 local healthcare systems that began in phase 1 ○ Bachelor’s Degree training, little/no previous exposure to quality improvement methods ○ Trained in QI by and support for data/measures from VAIL HSRs ● Rapid reviews of literature pertaining to QI project topics [24] ● Voluntary participation in/use of data/measures support group bi-weekly meetings, and privacy/ethics reviews of QI project activities ● A SharePoint site for housing toolkits from successful QI projects, support for toolkit development	Proportion of projects using data to diagnose QI problem and track QI project feasibility/acceptability/effectiveness for all approved projects: high = used evidence/data to diagnose QI problem and track progress for 100% of projects; medium = used evidence/data to diagnose and track for more than 60% of QI projects; low = used evidence/data to diagnose and track for 60% or less of QI projects.
**EBQI-PCMH Outcome—implementation and spread of locally developed and initiated QI projects**	
● Implement Steering Committee approved projects, using Plan-Do-Study-Act cycles ● Complete interim and final reports with data to track/monitor progress on achieving QI project objectives ● Briefings, presentations at QI collaborative in-person learning sessions to promote adoption by other sites ● Package QI project tools and materials into toolkits, with assistance from the VAIL project staff	Number of final reports + toolkits completed: high = 4 or more; medium = 2–3; low = 1; none = 0

## Methods

### Setting and participants

Using a modified stepped-wedge design [49] with three phases [19, 20], EBQI-PCMH was introduced in five local healthcare systems in the Southern California/Nevada region from April 2010 to September 2014. Participants included multi-level, interdisciplinary leaders, primary care providers and staff, other clinicians (e.g., social workers, pharmacists, behavioral health), health services researchers, VAIL project staff, and patient representatives. In phase 1 (April 2010–May 2011), one primary care practice at each of three local healthcare systems began participating in EBQI-PCMH activities. Three additional primary care practices from the same 3 healthcare systems began participating in phase 2 (January 2012). In phase 3 (September 2013–January 2014), we added one primary care practice from one of the original three healthcare systems and two primary care practices from two new healthcare systems. By the end of phase 3, five local healthcare systems (nine primary care practices) and seven across-site, topic-focused workgroups composed of VHA and non-VHA subject matter experts had participated in EBQI-PCMH. Participating primary care practices included four large medical center-based clinics (16,000 or more unique primary care patients) and five medium-large community-based outpatient clinics (8000–20,000 unique primary care patients). The initiative also included a Steering Committee composed of regional and local healthcare system executive leaders.

### Data sources and measures

We used project administrative records, systematically reviewing them and abstracting data to construct fidelity measures for the EBQI elements described in Table 1, and a measure to assess the EBQI short-term outcome of implementation and spread of successful locally developed and initiated QI projects. The following procedures were followed to ensure the accuracy of data abstraction. A primary reviewer reviewed the administrative documents, abstracted information, and entered the information into a database with pre-specified fields. A secondary reviewer reviewed the same documents and checked them against the database to assure the accuracy of information entered. Errors or disagreements were discussed with the lead author, who made final decisions about values assigned. Supplemental Table 1 contains a complete list of variables derived from administrative records.

We mapped data extracted from administrative records to activities corresponding to each of three EBQI core elements and the outcome and created site/workgroup-level measures (Table 1). Based on the distribution of these measures, we developed and applied specific criteria to determine high, medium, and low/no fidelity. We chose cut-points based on how the values clustered, taking into account high outliers. For example, for the sum of number of QI projects proposed and approved, sites A and B were clear outliers with 21 and 19 respectively, while sites C and D were the next highest with 8. We set the cut-point for high at 8 to conservatively include the high outliers and the next highest achieving sites. (See Supplemental Table 2 for data for each site and workgroup.)

Of note, we did not have complete administrative records for across-site, topic-focused workgroup participation. These groups did not routinely participate in bi-weekly collaborative calls. Most did organize and hold their own regularly occurring meetings supported by VAIL staff, but we were unable to locate minutes or other records from these meetings. We were also missing 5 months of meeting minutes for the bi-weekly collaborative calls. We report in Supplemental Table 2 that VAIL organized 87 bi-weekly conference calls, when in fact the total was around 100 including these 5 months.

To understand the researchers’ roles in variations in fidelity, we analyzed 73 qualitative interviews from regional (7), healthcare system (22), and primary care practice (21) leaders; other site participants, including Veteran patient representatives (8); VAIL team members (12); and internal coordinators (8). Interviews were conducted in Sept 2013–Feb 2014 and July 2014–Jan 2015, with 51/60 (88%) and 22/27 (92%) agreeing to participate, respectively. Ten key stakeholders were interviewed in both waves and 53 were interviewed only once. Interviews were semi-structured, most were conducted in-person, and lasted approximately 60 min. Audio recordings were professionally transcribed, reviewed by the research team, and edited for accuracy. Interviews contained questions about EBQI-PCMH features, including VAIL Steering Committee experiences, internal coordinators, support and resources received from the VAIL project team, and collaborative learning sessions.

### Analysis

To assess fidelity for EBQI-PCMH core elements and implementation and spread of locally initiated QI projects, we applied criteria described in Table 1 for high, medium, or low/no fidelity to the measures.

To analyze the interview data, the lead author developed an initial code list of a priori codes pertaining to core elements of the EBQI-PACT implementation strategy. She and a second qualitative analyst used the code list to independently code the same five interviews; discrepancies in applying codes were discussed and definitions of each code were refined to create a codebook. Using this codebook and Atlas.ti, a trained qualitative analyst coded all interviews, and a second qualitative analyst reviewed all coding. We then generated Atlas.ti reports for the codes for VAIL Steering Committee, collaborative learning sessions, VAIL team support and resources, quality improvement projects, and internal coordinator, and used matrix analysis to identify common themes related to each EBQI-PCMH core element. Specifically, the lead author did the following: (1) abstracted data from the reports and entered quotes/paraphrased quotes from each interview into fields in an Excel spreadsheet corresponding to the core elements, and (2) summarized experiences described across multiple interviewees into themes. A second qualitative analyst reviewed the data in the spreadsheet to confirm the themes. While rare, disagreements about coding, application of codes, and derivation of the themes were resolved through discussion and consensus [50].

## Results

Data for fidelity measures and the outcome appear in Tables 2, 3, and Supplemental Table 2. Duration of participation in EBQI-PCMH (Supplemental Table 2) was similar across sites within implementation phase, except for sites C and G which lagged slightly behind. Duration of participation could not be calculated for 5 across-site workgroups, because either they had no approved QI projects or we had incomplete administrative data for those groups.

**Table 2:** EBQI-PCMH fidelity for participating primary care practice sites

	Phase 1 (87 meetings, 7 conferences)			Phase 2 (60 meetings, 5 conferences)			Phase 3 (18 meetings, 1 conference)		
	Site A	Site B	Site C	Site D	Site E	Site F	Site G*	Site H	Site I
Leadership and frontlines (e.g., top-down, bottom-up) priority-setting process for focusing QI efforts									
Sum number QI projects proposed + approved	High	High	High	High	Med	Med	Med	Low	Low
Ongoing technical expertise and coaching/mentoring in QI methods by health services researchers, delivered through a QI collaborative									
Bi-weekly QI collaborative calls with representation	Med	Med	High	Low	Med	High	Med	None	None
Number of representatives attending learning sessions	High	High	High	Med	High	Med	None	Med	Med
Use of data and evidence to inform QI efforts									
Reported using evidence/data to ID problem and track progress	High	Med	Med	Low	High	High	Low	Low	Low
EBQI-PCMH change mechanism: implementation and spread of locally developed and initiated QI projects									
Number of final reports + toolkits completed	High	High	High	Med	Med	Med	Med	None	None

*Site G did not begin participating in EBQI-PACT until January 2014, after the last collaborative conference (Sept 2013)

**Table 3:** EBQI-PCMH fidelity for across-site, topic-focused workgroups

	WG 1	WG 2	WG 3	WG 4	WG 5	WG 6	WG 7
Leadership and frontlines (e.g., top-down, bottom-up) priority-setting process for focusing QI efforts							
Sum number QI projects proposed + approved	Med	Low	Med	Med	Low	Low	Low
Ongoing technical expertise and coaching/mentoring in QI methods by health services researchers, delivered through a QI collaborative							
Bi-weekly QI collaborative calls with representation	None	None	None	None	None	None	None
Number of representatives attending learning sessions	Med	Low	High	Med	Med	Med	Low
Use of data and evidence to inform QI efforts							
Reported using evidence/data to ID problem and track progress**	High	NA	High	Low	NA	NA	NA
EBQI-PCMH change mechanism: implementation and spread of locally developed and initiated QI projects							
Number of final reports + toolkits completed	Low	None	Med	Med	None	None	None

**NA in this row indicates the workgroup had no approved projects

### Leadership and frontline priority-setting process for primary care QI

#### Site/workgroup fidelity

As shown in Tables 2 and 3, 7 of 9 sites and 3 of 7 across-site workgroups achieved high or medium fidelity. Sites and workgroups submitted 72 QI project proposals over 4 years (Supplemental Table 2). Twenty-four regional and healthcare system leaders served as members of the VAIL Steering Committee and reviewed and rated proposals across four rounds of priority-setting. They approved 26 projects to receive VAIL support. Sites with longer duration in the project (e.g., phase 1 and 2 sites) had higher fidelity for this element.

#### Health services researchers’ role

VAIL health services researchers and staff organized the priority-setting process, including arranging meeting logistics, assisting sites and workgroups with preparing QI project proposals, and developing rating forms and a process for reviewing and approving projects. Engaging both leaders and frontlines in priority setting facilitated bi-directional communication and built consensus around which projects to prioritize. Key stakeholders at all sites noted that regional and executive-level leaders signaled organizational priorities through their approval of QI projects. As one site participant described, “It was good for me to see how engaged our leadership is… It helped me see the [leaders] as more of a support role instead of a role where people are like no, you can’t do this, trying to figure out how we can make things happen.” Inclusion of frontline providers and staff in priority-setting ensured that leaders learned about PCMH implementation challenges and successes. As one regional leader explained, “I’m really getting the ear from the Steering Committee members [about] what is going on at facilities and the forward movement…being able to work together and share opinions on what areas are important and what areas aren’t has been really helpful and a really powerful way to move forward.”

Feedback from a few key stakeholders indicated that some may not have fully understood the purpose of the priority-setting process and/or it was not sufficiently transparent: “So VAIL and the Steering Committee seem to have…got a little bit politicized over time…and degenerated significantly, in my opinion” (site participant). A key stakeholder at one of the less-engaged sites thought that allowing sites (rather than the Steering Committee) to determine which projects they implemented would foster more “ownership” and incentivize participation.

### QI learning collaborative for QI teams

#### Site/workgroup fidelity

Learning collaborative activities included participation in bi-weekly phone calls and in-person learning sessions. As shown in Tables 2 and 3, only 6 of 9 sites (and no across-site workgroups) achieved high or medium fidelity for bi-weekly phone calls. Participation in learning sessions was generally high, with all but one site and one workgroup attending at least one, and most attending all 7 (Supplemental Table 2). Number of participants per site or workgroup varied between 1 and 28. In terms of leadership participation, site primary care practice leaders participated regularly in calls, but regional and executive healthcare system leaders only participated in in-person learning sessions (15–47 participants per learning session, 33–50% of all attendees). Four Veteran patient representatives also participated in learning sessions.

#### Health services researchers’ role

The VAIL team organized and participated in bi-weekly calls and twice-yearly in person learning sessions as part of the QI collaborative for sites/workgroups with approved QI projects. Health services researchers mentored and coached the QI teams in QI methods, and moderated discussion between participants. Key stakeholders reported that collaborative activities were helpful for fostering innovation, contributed to a sense of community, and provided reassurance that everyone was experiencing the same challenges. As one site participant described, “Every single last person that’s come [back] from a collaborative meeting has said, ‘Gosh, I wish we had more time to talk to people from the sites.’ Not chitchat or gossiping, it’s about actual quality improvement, we want to ask specific questions and get specific answers and that takes a certain forum to be able to do that.”

The QI collaborative activities set the expectation for cross-site sharing and innovation spread, promoting regional development of a QI culture: “If VAIL went away, somebody would have do some of the things VAIL is doing because otherwise, it’s going to be, oh, [healthcare system]’s over here doing whatever they’re doing and [healthcare system] won’t share with anybody…having VAIL forced us to come to the sandbox and play together” (site participant). Many key stakeholders reported that QI collaborative activities reinforced local mechanisms for QI oversight and accountability by setting the expectation of regular reporting on calls and at conferences. As a VAIL team member explained, “having that level of leadership engaged in the initiative has really made people more accountable. It really conveys to them the sense that the [regional leadership] is taking [this] very seriously.”

Comments from some key stakeholders suggested that a basic level of “readiness” may be necessary for sites to prioritize collaborative activities. A few key stakeholders, for example, questioned the utility of focusing on QI when more basic needs at their sites had not been addressed: “Sometimes I feel like it’s too grandiose of a plan when basic resources are missing, that you can’t implement things if you don’t have certain pieces like staff.” One key stakeholder was not sure how useful the QI toolkits really were, because they may not work or need a lot of adaptation due to variation in how PCMH teams were configured at different sites. In addition, a few key stakeholders noted that getting release time for QI team members to attend in-person meetings was difficult.

### Technical assistance for using data and evidence for QI

#### Site/workgroup fidelity

As shown in Tables 2 and 3, fidelity for this element was mixed, and does not appear to be associated with QI implementation and spread or duration of participation in EBQI-PCMH. Except for site G, sites and across-site workgroups with low fidelity for use of data also achieved low fidelity for bi-weekly calls, suggesting they may not have been well-connected with VAIL resources and supports.

#### Health services researchers’ role

VAIL health services researchers supported use of evidence and data by providing rapid evidence reviews, formative feedback on sites’ performance, and help with obtaining data and evaluating QI project impact. Key stakeholders reported that the VAIL team and internal site coordinators were helpful for obtaining data and creating measures to track their QI projects and for general PCMH improvement efforts. The expectation that QI teams would collect and report data on feasibility and effectiveness facilitated forward momentum, because “measurement keeps people honest in a way, or it gives a reality check. Because people can say things are happening, but if it’s not documented and measured, it really didn’t happen” (site participant). The internal coordinators described their role as number crunchers, problem solvers, liaisons with leadership and the VAIL project, and quality improvement coaches/teachers for PCMH teams, and key stakeholders at all sites considered them essential. Key stakeholders recalled that internal coordinators organized meetings, provided support for QI projects, and provided data and reports for improving PCMH performance. Commenting on reports provided by the internal coordinator, one site participant explained, “It’s really frustrating when you get a number that doesn’t seem to reflect reality…you get an A-plus if you look at third next available, but then you get an F when you’re looking at the Compass and you’re thinking how is this possible?… If they took away our coordinator I would drown.”

For a few sites, stakeholders reported that internal coordinators played a critical role as liaisons with the VAIL team, receiving assistance from VAIL for data and measures on their site’s behalf and reporting on the site’s activities. Coordinators participated in QI collaborative activities, and were frequently the only representatives for sites B, D, and E on bi-weekly calls. A few sites temporarily or permanently lost the support of their internal coordinators. Key stakeholders at those sites reported difficulty obtaining accurate and reliable data for QI, lapses in or discontinuation of meetings, and losing their connections with the VAIL team. After losing their coordinator, one site B participant lamented, “That individual essentially held the [site-level QI] group together. Once you bring them together, you have your best chance of coming up with ideas and then enforcing them. And once that’s gone, we don’t even meet. So how can we come up with a project let alone continuing and reporting to make sure it does well? It’s all gone.”

Some key stakeholders were not aware of resources or support provided by VAIL, and suggested that an organizational chart of the VAIL project would be helpful. One site, comparing themselves to another site, felt they could have been more successful if they had more direct access to the VAIL team: “I don’t know where VAIL fits in without having that research person there or [internal] coordinator who really understands how to frame it.”

### EBQI-PACT outcome: implementation and spread of successful locally developed and initiated QI Projects

As of the end of the study period (October 2014), sites and across-site workgroups had completed 21 projects, with 16 resulting in tools/toolkits for spread (Supplemental Table 2). As shown in Tables 2 and 3, phase 1 sites completed the most projects and toolkits. Two phase 3 sites and four workgroups did not complete any projects or toolkits. Generally, sites with a longer duration were more successful implementing and spreading their QI projects. Workgroups were less successful; only 3 workgroups completed projects and/or toolkits. Sites and workgroups with the highest levels of participation in priority-setting and collaborative learning sessions appear to have had more success.

## Discussion

This study advances implementation theory and research by developing measures for and assessing fidelity to the EBQI implementation strategy as applied to PCMH. Translating a successful intervention into routine practice requires use of effective implementation strategies that are appropriate to the context, but development of a knowledge base about which implementation strategies “work” and under what conditions has been hampered by inconsistent use of terminology, lack of operational definitions, and poorly defined fidelity measures [51, 52]. Implementation researchers highlight the importance of defining, describing, and evaluating fidelity to implementation strategies in order to bridge the evidence to practice gap. Previously published impact analyses of EBQI-PCMH demonstrated its overall effectiveness for accelerating achievement of key PCMH goals within VHA [21–23]. In previous papers [19, 20], we described the theoretical origins and presented a logic model hypothesizing that key features of EBQI-PCMH facilitate achievement of PCMH goals through the implementation and spread of effective QI projects. Our findings here highlight three core elements of EBQI-PCMH as “active ingredients” that, in combination, facilitated QI efforts to adapt PCMH to local contexts and spread innovation across sites, helping EBQI-PCMH sites better achieve PCMH goals.

Results from this study have important theoretical and practical implications for PCMH implementation. First, we demonstrate how PCMH implementation can be improved by using EBQI-PCMH to target barriers to change at multiple levels. At the organizational and provider/staff levels, EBQI-PCMH engaged multi-level, interdisciplinary leaders and frontline primary care clinicians and staff in priority-setting for primary care. Our fidelity assessment found high to medium fidelity for this core element, with higher fidelity associated with longer participation in the overall initiative. EBQI-PCMH fostered bi-directional communication across hierarchical and disciplinary boundaries and set expectations for collaboration across service lines, reporting, and accountability. By identifying the challenges that different primary care practices may encounter with PCMH implementation at different stages, the priority-setting process also allowed individual practices to tailor PCMH implementation to address contextual factors at their sites. Although one previous study found successful PCMH implementation to be associated with empowering/authorizing leaders to make change [14], ours is the first study that we know of that has documented and assessed leadership engagement in QI priority-setting as an implementation strategy to improve PCMH implementation.

Second, our results support findings from a previous study of the American Academy of Family Physicians’ National Demonstration Project that showed PCMH adoption was associated with practice facilitation, participation in a learning collaborative, and ongoing consultation [13, 15]. Our study and previous EBQI studies describe ongoing technical expertise and coaching/mentoring in QI methods, provided by health services researchers, as a core element of the strategy [34, 53]. EBQI-PCMH applied this primarily through an organized QI learning collaborative with bi-weekly calls and semi-annual in-person learning sessions, as well as individual consultations with QI teams as needed/requested. Our learning collaborative provided QI teams with opportunities for cross-site sharing and learning, fostering a regional culture of QI, and promoting idea-generation and innovation. Regular reporting by QI teams in these forums also enforced accountability of QI. We posit that this learning and QI culture permeated EBQI-PCMH sites and may have changed their approach to addressing challenges with PACT implementation. Practice leaders were now empowered with knowledge of QI methods and techniques (as well as what has been tried at other primary care practices) and may have applied this learning more broadly to improve primary care delivery in their clinics.

Third, as the “learning healthcare system” gains popularity as a means of improving efficiency and quality and reducing costs [54, 55], healthcare systems will need to develop “health data infrastructures” capable of extracting and transmitting evidence and knowledge to inform decision-making [55, 56]. Systematic translation and dissemination of what is learned will also require application of effective implementation strategies [57]. The clinical-researcher partnership that provides the foundation for EBQI-PCMH highlights the role of health services researchers in transformative initiatives such as PCMH implementation. In particular, use of data and evidence to inform QI innovation (core element 3) is foundational to the EBQI-PCMH implementation strategy [37, 53], and was found in other studies to be important for successful PCMH implementation in small practices (e.g., as used for audit and feedback, conducting small tests of change) [14]. Our qualitative data suggests that support for using data and project management provided by the VAIL team and site internal coordinators was considered crucial to QI project successful completion, as well as general primary care QI efforts. The mixed levels of fidelity for this core element may indicate that our fidelity measures did not fully capture the impact of ongoing exposure to data and measurement expertise particularly through the internal coordinators. The coordinators may have served as knowledge brokers because they were hired by and worked directly for the sites but were mentored by the VAIL team [58].

Our results also highlight several areas for future research on implementation strategies. In combination with our previous studies showing accelerated PCMH implementation in EBQI-PCMH sites, this study suggests that a multi-faceted implementation strategy that combines leadership-frontline engagement in primary care QI; support, mentoring, and training for QI delivered via a QI collaborative; and, technical support for using data/evidence for QI and project management can improve implementation. A critical knowledge gap still exists, however, in terms of linking implementation strategy fidelity to site-specific outcomes (for example, does fidelity to using data to inform QI translate into better patient experience outcomes?). For this, researchers should focus on developing and validating implementation strategy fidelity measures that can be reliably and systematically collected across a variety of PCMH settings. These fidelity measures could then be tested in type 3 hybrid type studies with larger samples (e.g., more sites) [59].

Our results have several practical implications for healthcare policy makers, administrators, and leaders seeking to implement or improve uptake of PCMH in primary care. First, sites with longer exposure to EBQI-PMCH had higher fidelity to its core elements and were more successful with QI implementation and spread, suggesting that development of a QI culture may take a few years to achieve. Second, primary care practices in general had few if any resources to support QI, and thus the role of the internal coordinator dedicated to primary care QI will be important for organizations considering using this implementation strategy. Third, in addition to someone who can act as an internal coordinator to assist with QI efforts, embedded health services researchers [60] can improve PCMH implementation by working with existing personnel (including the internal coordinator) within the site to guide implementation efforts, using data/evidence to inform implementation, and providing mentoring/training in QI methods. Finally, as a large integrated healthcare system with a well-developed electronic health record and performance reporting system, VHA was an ideal setting in which to use EBQI-PCMH. Smaller healthcare systems or practices lacking the ability to easily obtain and analyze data on their care delivery processes and patient outcomes may need to budget more time and resources to support using data to inform QI, and should consider hiring/assigning a dedicated internal coordinator to help with this aspect. Payors could also provide primary care practices with access to data on each practices’ patients and incentivize use of EBQI in other ways. Professional societies could provide opportunities, such as QI learning collaboratives, for cross-site learning and sharing among small practices and/or healthcare systems.

### Limitations

The study had limitations. First, lack of data for across-site workgroup meetings may have affected the validity/reliability of our fidelity assessment for participation in the QI learning collaborative (core element 2) for these groups. Fidelity for workgroups, however, was low across all three EBQI-PCMH elements, suggesting that more could have been done to support their QI project efforts, and/or that cross-site topic-focused workgroups may not be an effective mechanism for facilitating local primary care QI. Missing administrative records for 5 months of bi-weekly calls that impacted fidelity measures equally for all sites and workgroups also resulted in an underreporting of participation (and resources devoted to participating) in EBQI-PCMH core element 2. Additionally, we did not systematically collect data on how sites were selected by healthcare system leaders, but we heard (informally and in interviews) from healthcare system leaders that they selected sites for a variety of reasons, including readiness (for example, one phase 1 site had residents who were required to conduct QI projects), resources and staffing, and sites that they thought might need extra help with PCMH implementation. Furthermore, we have anecdotal reports of sites conducting QI projects that were proposed but not approved by the VAIL Steering Committee, but we did not track or collect data on these additional QI projects and may have underreported the number of initiated and completed QI projects. In addition, we did not interview key stakeholders at sites G, H, and I or some of the workgroups, and thus the findings from qualitative interviews may not fully represent experiences of sites with shorter duration of participation. Finally, EBQI-PCMH required substantial resources, but subsequent applications in VHA Women’s Health and a separate VHA-funded care coordination QI demonstration are testing how EBQI can be accomplished with fewer resources [53, 61].

Several study strengths outweigh these limitations. First, we compiled administrative data from many and varied sources, representing study activities over nearly five years. We are not aware of any studies of PCMH implementation strategy fidelity with this length and breadth of data. The clinical-researcher partnership underlying EBQI distinguishes it from other implementation strategies that rely on a top-down implementation approach and highlights the health services researchers’ role in supporting fidelity to EBQI. We supplemented administrative records with qualitative interview data from key stakeholders, permitting exploration of the health services researchers’ role and how they were able to engage leaders and frontlines with many competing demands in conducting structured QI informed by evidence, and how the VAIL team could have better facilitated QI implementation and spread across sites. Finally, this study advances our understanding of which implementation strategies hold promise for successfully transforming primary care to a PCMH model.

## Conclusion

This study described three core elements of a multifaceted implementation strategy—evidence-based quality improvement or EBQI—and assessed fidelity to EBQI as used to implement VHA’s PCMH. The findings revealed that multi-level participation in priority-setting, EBQI collaborative learning sessions, and data/evidence use to inform QI are key features of the EBQI implementation strategy that can accelerate achievement of key PCMH goals. Furthermore, successful implementation and spread of local primary care QI was enhanced by the following: (1) systematically linking multi-level, interprofessional leadership to front-line innovators; (2) across-site communication and learning; and (3) availability of project management and data support. While this study has demonstrated that fidelity to implementation strategies can be assessed using a variety of data sources, to advance implementation science, future research should focus on the development of tools to systematically and prospectively measure and assess implementation strategy fidelity [62]. The practical implication of this study is that healthcare system leaders can incorporate key features of EBQI to improve implementation of evidence-based interventions.

## Supplementary information

**Additional file 1.** EBQI Core activities, Participation Measures, and Data Sources.

**Additional file 2.** Site and Across-site Workgroup Participation in Core EBQI-PACT Components.

## Data Availability

The datasets used and analyzed during the current study are available from the corresponding author on reasonable request. Supplemental Table 2 contains the data used for the fidelity measures reported in this paper.

## Abbreviations

EBQI: Evidence-based quality improvement
PACT: Patient-Aligned Care Team
PCMH: Patient-centered medical home
QI: Quality improvement
VAIL: Veterans Assessment and Improvement Laboratory
VHA: Veterans Health Administration
