# Supplementary material for: Assessing fidelity to evidence-based quality improvement as an implementation strategy for patient-centered medical home transformation in the Veterans Health Administration
Source: Implement Sci. 2020 Mar 18;15:18. doi: 10.1186/s13012-020-0979-y (PMC7079486; doi:10.1186/s13012-020-0979-y)
Supplement: Supplementary file 1 — Additional file 1. EBQI Core activities, Participation Measures, and Data Sources. [file 13012_2020_979_MOESM1_ESM.docx]

Supplemental Table 1. EBQI Core activities, Participation Measures, and Data Sources

| EBQI Core Activities | Participation Measures (by site/workgroup) | Administrative Data Sources |
| --- | --- | --- |
| Leadership and frontline priority-setting process for primary care QI | # QI projects proposed | QI project proposals |
|  | # QI projects approved | VAIL Steering Committee meeting materials (agendas, handouts) |
|  | # QI projects completed final report | QI project final reports |
|  | # Toolkits | QI project final reports, VAIL SharePoint site |
|  | # regional and healthcare system leaders that reviewed proposals | VAIL Steering Committee proposal review rating forms |
| QI learning collaborative for frontline primary care and cross-site QI teams | Duration of participation (in months) - approximate date that the site had: 1) an internal coordinator to provide support; 2) a primary care quality council that met at least once per month; and 3) at least one approved QI project. | Minutes of bi-weekly collaborative conference calls |
|  | # conference calls with representation | Attendance data from minutes of bi-weekly collaborative conference calls |
|  | Average # of representatives per call | Attendance data from minutes of bi-weekly collaborative conference calls |
|  | # in-person conferences with representation | In-person collaborative conference attendance rosters |
|  | Average # participants per in-person conference | In-person collaborative conference attendance rosters |
|  | % of participants at in-person conference who were regional, healthcare system, or local leaders | In-person collaborative conference attendance rosters |
|  | # patient representatives that attended in-person conferences | In-person collaborative conference attendance rosters |
| Technical assistance from health services researchers in the use of data and evidence | # QI projects using evidence/data to identify the QI problem | QI project proposals, interim reports, and final reports |
|  | # QI projects presented data in final report to VAIL Steering Committee | QI project final reports |
|  | # VAIL Health Services Researchers, statistical analysts, and program support staff | VAIL project staffing lists |

Table notes: QI project proposals were prepared by the site and workgroup QI teams with help from site internal coordinators and VAIL administrative staff, and used a standard template containing project title, problem, goals/aims, QI team member names, staff time and resources needed, measures and action plan, and timeline. Steering Committee proposal reviews included structured items with response choices to rate the proposals. QI project interim and final reports were completed by the site and workgroup QI project teams, and included structured items with response choices for project status and extent to which aims were achieved, and open-ended questions for project description, results, products, and spread. Conference call minutes, attendance rosters, and staffing reports were created by VAIL administrative staff and included participant names, sites, and dates.
