# Supplementary material for: Assessing fidelity to evidence-based quality improvement as an implementation strategy for patient-centered medical home transformation in the Veterans Health Administration
Source: Implement Sci. 2020 Mar 18;15:18. doi: 10.1186/s13012-020-0979-y (PMC7079486; doi:10.1186/s13012-020-0979-y)
Supplement: Supplementary file 2 — Additional file 2. Site and Across-site Workgroup Participation in Core EBQI-PACT Components. [file 13012_2020_979_MOESM2_ESM.docx]

Supplemental Table 2. Site and Across-site Workgroup Participation in Core EBQI-PACT Components

|  | Phase 1  (87 meetings, 7 conferences) | | | Phase 2  (60 meetings, 5 conferences) | | | Phase 3  (18 meetings, 1 conferences) | | |
| --- | --- | --- | --- | --- | --- | --- | --- | --- | --- |
|  | Site A | Site B | Site C | Site D | Site E | Site F | Site G | Site H | Site I |
| Participation in priority-setting process | | | | | | | | | |
| QI projects proposed | 16 | 16 | 5 | 5 | 5 | 2 | 2 | 1 | 1 |
| QI projects approved | 5 | 3 | 3 | 3 | 1 | 2 | 2 | 1 | 1 |
| Participation in QI collaborative | | | | | | | | | |
| Duration of participation (in months) | 50 | 48 | 41 | 32 | 32 | 32 | 9 | 14 | 14 |
| % QI collaborative calls with representation | 86%  (75) | 86%  (75) | 93%  (81) | 25%  (15) | 85%  (51) | 95%  (57) | 89%  (16) | 0 | 0 |
| Ave. # representatives per QI collaborative call | 1.6 | 1.7 | 3.0 | .3 | 1.5 | 2.8 | 1.7 | 0 | 0 |
| # QI learning sessions with representation | 7 | 7 | 7 | 7 | 7 | 7 | 0 | 1 | 1 |
| # of representatives attending learning sessions | 28 | 21 | 12 | 7 | 18 | 8 | 0 | 7 | 9 |
| QI projects use of evidence/data for approved projects | | | | | | | | | |
| Reported using evidence/data to identify the QI problem | 100%  5 | 67%  (2) | 67%  (2) | 60%  (3) | 100%  (1) | 100%  (2) | 100%  (2) | 100%  (1) | 100%  (1) |
| Presented data in final report | 100%  (5) | 100%  (3) | 67%  (2) | 20%  (1) | 100%  (1) | 100%  (2) | 0 | 0 | 0 |
| EBQI-PCMH Outcome - implementation and spread of locally developed and initiated QI projects | | | | | | | | | |
| QI projects completed final report | 5 | 3 | 3 | 1 | 1 | 2 | 2 | 0 | 0 |
| Toolkits | 2 | 2 | 1 | 1 | 1 | 1 | 0 | 0 | 0 |

|  | Across-site Workgroups | | | | | | |
| --- | --- | --- | --- | --- | --- | --- | --- |
|  | WG 1 | WG 2 | WG 3 | WG 4 | WG 5 | WG 5 | WG 6 |
| Participation in priority-setting process | | | | | | | |
| QI projects proposed | 2 | 2 | 3 | 6 | 1 | 2 | 2 |
| QI projects approved | 1 | 0 | 2 | 2 | 0 | 0 | 0 |
| Participation in QI collaborative | | | | | | | |
| Duration of participation (in months)^a^ | No data | No data | 45 | 45 | No data | No data | No data |
| % QI collaborative calls with representation | 0 | 0 | 0 | 2 | 0 | 0 | 0 |
| Ave. # representatives per QI collaborative call | 0 | 0 | 0 | .03 | 0 | 0 | 0 |
| # QI collaborative conferences with representation | 6 | 0 | 7 | 7 | 6 | 6 | 6 |
| # representatives attending learning sessions | 9 | 0 | 12 | 7 | 6 | 5 | 1 |
| QI projects use of evidence/data for approved projects | | | | | | | |
| Reported using evidence/data to identify the QI problem | 100%  (1) | NA | 100%  (2) | 100%  (2) | NA | NA | NA |
| Presented data in final report | 100%  (1) | NA | 100%  (2) | 50%  (1) | NA | NA | NA |
| EBQI-PCMH Outcome - implementation and spread of locally developed and initiated QI projects | | | | | | | |
| QI projects completed final report | 0 | 0 | 2 | 2 | 0 | 0 | 0 |
| Toolkits | 1 | 0 | 0 | 1 | 0 | 0 | 0 |

^a^ We did not have sufficient data to assess duration of participation for most of the across-site workgroups. For the two workgroups that submitted QI projects for all 3 rounds of priority-setting, we estimated the start date to be the date of Steering Committee approval of their first projects, with duration lasting until the end of the evaluation period (September 2014).

Table notes: Site G did not begin participating in EBQI-PACT until January 2014, after the last collaborative conference (Sept 2013).
